# The Protective Effects of Zeaxanthin on Amyloid-β Peptide 1–42-Induced Impairment of Learning and Memory Ability in Rats

**DOI:** 10.3389/fnbeh.2022.912896

**Published:** 2022-06-23

**Authors:** Xiaoying Li, Ping Zhang, Hongrui Li, Huiyan Yu, Yuandi Xi

**Affiliations:** ^1^Department of Geriatrics, Beijing Jishuitan Hospital, Beijing, China; ^2^Department of Nutrition and Food Hygiene, School of Public Health, Capital Medical University, Beijing, China

**Keywords:** zeaxanthin, amyloid-β peptide 1–42, learning and memory ability, oxidative stress, inflammation, cerebrovascular

## Abstract

**Background and Objectives:**

Zeaxanthin (ZEA) as one of the biologically active phytochemicals presents a neuroprotective effect. Since ZEA may play its anti-oxidative role in neurodegenerative diseases including Alzheimer’s disease (AD), we hypothesized cognitive defects could be prevented or deferred by ZEA pre-treatment.

**Methods and Study Design:**

All the rats were randomly divided into four groups (control, Aβ1–42, ZEA, and ZEA + Aβ groups). Learning and memory ability of rats, cerebrovascular ultrastructure changes, the redox state, endothelin-1 (ET-1) level, and amyloid-β peptide (Aβ) level in plasma and the Aβ transport receptors which are advanced glycation end products (RAGEs) and LDL receptor-related protein-1 (LRP-1) and interleukin-1β (IL-1β) expressions in the cerebrovascular tissue were measured in the present study.

**Results:**

The escape latency and frequency of spanning the position of platform showed significant differences between the Aβ group and ZEA treatment groups. ZEA could prevent the ultrastructure changes of cerebrovascular tissue. In addition, ZEA also showed the protective effects on regulating redox state, restraining ET-1 levels, and maintaining Aβ homeostasis in plasma and cerebrovascular. Moreover, the disordered expressions of RAGE and LRP-1 and IL-1β induced by Aβ1–42 could be prevented by the pre-treatment of ZEA.

**Conclusion:**

ZEA pre-treatment could prevent learning and memory impairment of rats induced by Aβ1–42. This neuroprotective effect might be attributable to the anti-oxidative and anti-inflammatory effects of ZEA on maintaining the redox state and reducing the Aβ level through regulating the Aβ transport receptors and inflammatory cytokine of the cerebrovascular tissue.

## Introduction

Cognitive decline is the common clinical behavior change in Alzheimer’s disease (AD) (Scheltens et al., 2021). Amyloid-β peptide (Aβ) accumulation in the brain in patients with AD is regarded as the main pathological feature of AD (Qiang et al., 2017). Recent studies have shown that atherosclerosis (Lin et al., 2022), cerebrovascular endothelial damage (Xi et al., 2012), the Aβ clearance dysfunction (Xi et al., 2013), the oxidative damage resulting in the blood–brain barrier (BBB) broken, and cerebral microvascular pathology (Boese et al., 2020; Kurz et al., 2021; Owens et al., 2022) have a close relationship with the advancement of AD. These findings suggest that cerebrovascular dysfunction might be key pathogenesis in the development of AD (Li et al., 2021). It is reported that oxidative damage is an important case in the progress of cerebrovascular pathology (Chen et al., 2022; Martini et al., 2022). Evidence showed the toxicity of Aβ could lead to cerebrovascular oxidative damage (Xi et al., 2014). Therefore, protecting the cerebrovascular system by antagonizing Aβ-induced oxidative damage might be a critical way to prevent or defer the progress of AD.

Zeaxanthin (ZEA), one of the most common carotenoids in nature, can be found in many vegetables for human daily consumption. Extensive epidemiological observations indicate that fruits and vegetables rich in carotenoids provide a variety of health benefits (Monjotin et al., 2022). It has been reported that the dietary carotenoids may be of benefit in maintaining cognitive health (Monjotin et al., 2022) and a high level of lutein in diet and serum may play a protective role in atherosclerosis (Wang et al., 2018). A study indicates that the consumption of lutein- and zeaxanthin-rich vegetables were associated with a lower rate of age-related cognitive decline (Martínez García et al., 2018; Liu et al., 2021). In addition, the level of zeaxanthin in plasma is inversely related to the risk of AD (Qu et al., 2021). It is well known that oxidative stress and inflammation play a key role in AD risk (Zhang et al., 2022). It is demonstrated that zeaxanthin has anti-oxidative and anti-inflammatory effects on many cell lines (Jia et al., 2017; Ge et al., 2020). Carotenoids including ZEA are also reported to have anti-amyloidogenic potential which can inhibit Aβ aggregation (Lakey-Beitia et al., 2017, 2019). However, it is still not clear about the protective role of zeaxanthin on cognitive ability and its mechanism. Therefore, we hypothesized that ZEA could prevent Aβ-induced impairment of learning and memory ability by reducing the Aβ in plasma and cerebrovascular tissue, this beneficial effect might be related to mediating the dysfunction of Aβ transportation and vascular oxidative stress. In order to test the hypothesis, we assessed the changes of learning and memory ability of rats, Aβ expression in plasma and neurovascular tissue, endothelin-1 (ET-1) level, glutathione (GSH), glutathione disulfide (GSSG), GSH/GSSG ratio in plasma, the receptor for advanced glycation end products (RAGEs) and LDL receptor-related protein-1 (LRP-1) expressions and inflammatory cytokine interleukin-1 (IL-1β) in the cerebrovascular tissue of rats after treating with or without ZEA and Aβ.

## Materials and Methods

### Experimental Animal

In total, forty-eight male Wistar rats (SPF class, age of 8 weeks, weighing 250–300 g, provided by the Academy of Military Medical Sciences) were divided into four groups randomly according to the body weights: control group, Aβ group, ZEA + Aβ group, and ZEA group. All the surgical procedures and animal handling were conducted in accordance with the Chinese Committee of Experimental Animal Supervision (Reg. No. AEEI-2014-047).

### Animal Treatment

**FIGURE 1:**
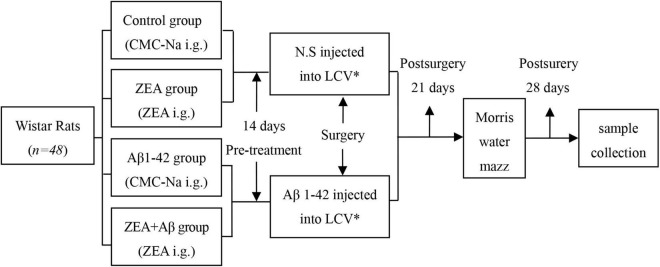
The schedule of the rat’s experiment. *LCV is lateral cerebral ventricle.

Different groups of rats were treated with sodium carboxymethyl cellulose (0.5% CMC-Na, control, and Aβ groups) or 60 mg/kg⋅d ZEA (ZEA lyophilized from 0.5% CMC-Na, ZEA, and ZEA + Aβ groups) by intragastric administration (i.g.) for 14 days. Then, surgery was taken to inject the solution of 0.9% w/v of NaCl (normal saline, N.S) or Aβ1-42 (A9810, Sigma-Aldrich, United States, dissolved in N.S., 20 μg/200 μl) into lateral cerebral ventricle by miniosmotic pump (Xi et al., 2015). Synthetic Aβ1–42 was dissolved in physiological saline at 0.1 mg/ml and incubated at 37°C for 3 days to form Aβ1–42 aggregation (Xi et al., 2014). The Morris water maze was measured on day 21 after surgery. Animals were executed, and the blood and brain tissue samples were collected on day 28, after surgery (Figure 1). The component parameters of ZEA (Lot: 10070701, provided by Innobioactives Limited, Dalian, China) are UV content 97.2% and HPLC composition including ZEA 90.0, lutein 8.2, and other carotenes 1.8%.

### Surgery Procedure

After rats were anesthetized by intraperitoneal injection of 10% sodium pentobarbital (1.3 g/kg body weight), vehicle (200 μl N.S, control, and ZEA groups) or Aβ1–42 solution (20 μg dissolved in 200 μl N.S, Aβ, and ZEA + Aβ groups) were injected by miniosmotic pump (Alzet 2002; Durect Co., Cupertino, CA, United States), respectively, into the targeted cerebral ventricle by stereotaxic frame. The position of inject hole was at anteroposterior –1.3 mm from Bregma, mediolateral 1.8 mm, dorsoventral –3.4 mm.

### The Morris Water Maze

The learning and memory abilities of rats were measured by the Morris water maze, 21 days after surgery. Rats were dropped into a water maze from 4 different sites of bucket wall for 4 days, respectively, for training the rats to find the hidden platform in the first quadrant. The escape latency was tested on day 5. Then, on day 6, the hidden platform was withdrawn, and the times of target crossing were recorded.

### Electron Microscopy

For transmission electron microscope studies, the brain tissues were removed and fixed in 3% glutaraldehyde (Alfa Aesar, United Kingdom), 0.1 M phosphate buffer, and 1% osmium tetroxide. Ultrathin sections (∼70 nm) were cut with an ultramicrotome and collected onto copper grids. Ultrathin sections were stained using 4% uranyl acetate-lead citrate and photographed under an electron microscope (TEM; JEM-1230, Japan).

### Determination of Redox Status in Plasma

GSH and GSSG assay kit was used to test redox status in plasma in rats. GSH/GSSG was calculated according to the instruction.

### Measurement of Aβ and Endothelin-1 Levels in Plasma

Blood was collected by vacuum EDTA anticoagulant blood tubes and needles from the heart. Overall, 10% sodium pentobarbital was used to make rats anesthetized. The blood was centrifuged for 10 min by 1,000 rpm at 4°C. Supernatant liquid (plasma) was collected. Aβ and ET-1 levels in the plasma of rats were tested by an enzyme-linked immunosorbent assay (ELISA) kit. The experimental procedure was executed strictly according to the manufacturers’ instructions. In total, 450 nm of color reaction was measured by the Microplate Reader (TECAN, Infinite M200, Switzerland). Results were calculated and expressed as ng/g protein.

### Immunohistochemistry

At the end of the animal experiment, six rats in different groups were dissected. First, 37°C PBS and 4°C paraformaldehyde were used in perfusion in succession. After the perfusion, the dissected brains were immersed in 4% formaldehyde solution immediately. In total, 24 h later, the tissue was transferred into 70% ethanol and embedded with paraffin. The 20 μm paraffin sections were operated according to the peroxidase–antiperoxidase method. The image analysis software was used to analyze RAGE, LRP-1, and IL-1β (ABCAM, United Kingdom) expressions in different groups.

### Statistical Analysis

We used SPSS11.5 software to analyze the data. The statistical results were expressed as mean ± SD. One-way ANOVA was used as appropriate. Values of *P* < 0.05 were considered statistically significant.

## Results

### The Protection of Zeaxanthin on Learning and Memory Ability of Rats

**FIGURE 2:**
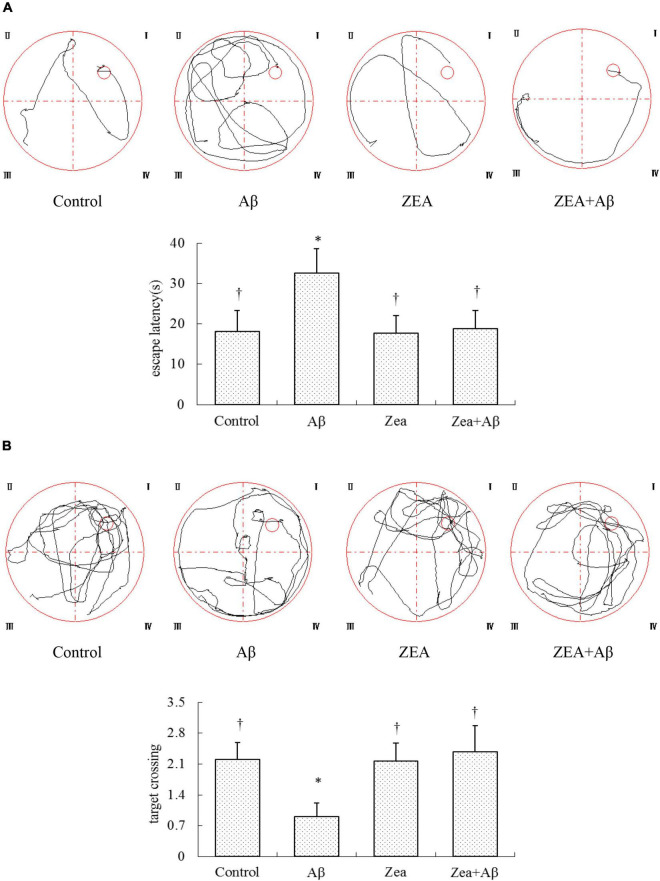
Neuroprotective effects of ZEA on escape latency **(A)** and frequency of rats crossing the position of the platform **(B)** in learning and memory impairment rats induced by Aβ1–42; Morris water maze test was taken from CMC-Na i.g. + N.S injected into lateral cerebral ventricle (LCV) group (Control group); CMC–Na i.g. + Aβ1–42 injected into the LCV group (Aβ group); ZEA i.g. + N.S injected into the LCV group (ZEA group); ZEA i.g. + Aβ1–42 injected into the lateral cerebral ventricle group (ZEA + Aβgroup) (*n* = 12 per group). All the data were shown as mean ± SD, and analyzed by the one-way ANOVA test [*post hoc* (LSD) test]. *Significantly different from the Control group (*p* < 0.05). ^†^Significantly different from the Aβ group (*p* < 0.05).

To explore the role of ZEA against learning and memory impairment induced by Aβ1–42, the Morris water maze was detected on day 21 after surgery. Escape latency in the rats of the Aβ group showed extremely prolonged than that in the control group. But in ZEA and ZEA + Aβ groups, the escape latency was significantly shorter compared with the Aβ group (Figure 2A). Similarly, the times of rats spanning the position of the platform are less in the Aβ group than that in the control group and two ZEA treatment groups (Figure 2B).

### The Maintenance of Zeaxanthin on the Ultrastructure of Cerebrovascular Tissue

**FIGURE 3:**
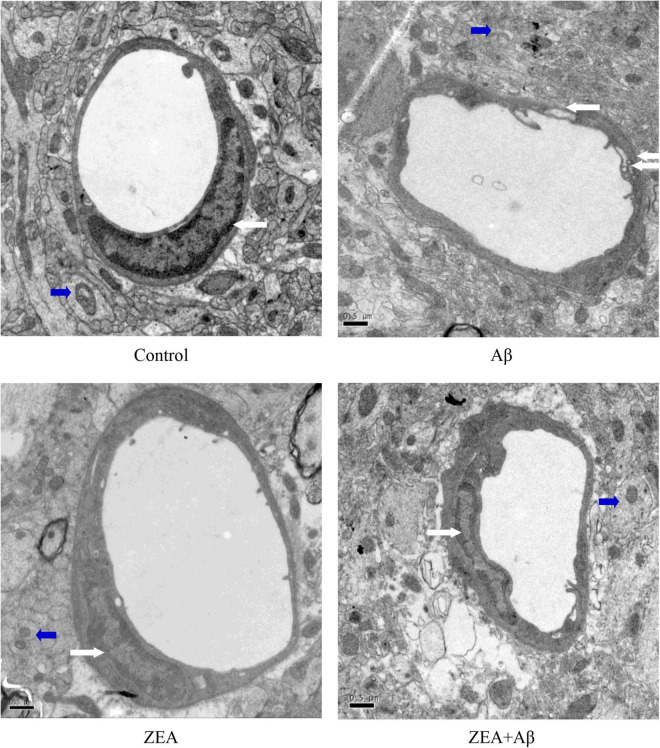
The ultrastructure (10,000 ×) of cerebrovascular (White Arrows) and brain tissue (Blue Arrows) in different groups. A transmission electron microscope was used in the section of the whole rat brain from our four treatment groups mentioned before. Staff gauge showed 0.5μm at the left side of every picture’s bottom.

In order to explore the morphological protection of ZEA on cerebrovascular and brain tissue, transmission electron microscopy was used in the present study. Figure 3 shows that in the control and ZEA groups, the structure of cerebrovascular endothelial cells was integrated and clearly visible. The mitochondria structure and shape of cerebrovascular endothelial cells in these two groups were normal. But in the Aβ group, cerebrovascular endothelial cells showed pathological changes including invisible structure and excess extracellular vesicles. Mitochondria swollen were also found in the Aβ group accompanied by vague/ruptured/melted mitochondrial cristae. However, in the ZEA + Aβ groups, the abnormal changes were obviously improved compared with the Aβ group. The same ultrastructure morphological protection of ZEA was also found in the neuron (Figure 3).

### The Restoration of Zeaxanthin on the Redox State of Rats in Plasma

**FIGURE 4:**
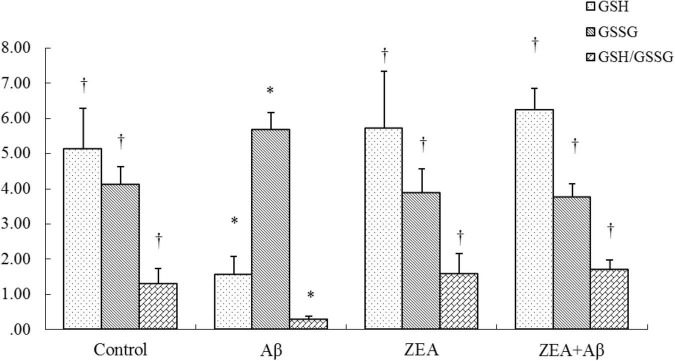
Neuroprotective effects of ZEA on GSH, GSSG, and GSH/GSSG in plasma of learning and memory impairment rats induced by Aβ1–42. The kit assay was used in rat plasma from four treatment groups mentioned before (*n* = 12 per group). All the data were shown as mean ± SD, and analyzed by the one-way ANOVA test [*Post Hoc* (LSD) test]. *Significantly different from the Control group (*p* < 0.05). ^†^Significantly different from the Aβ group (*p* < 0.05).

To determine whether ZEA could prevent the oxidative damage of rats induced by Aβ1–42, GSH, and GSSG, the sensitive indexes of body redox state were measured in plasma. The results showed that GSH level and GSH/GSSG ratio in plasma were significantly decreased in the rats of the Aβ group, while GSSG levels were dramatically increased compared with the control group. On the contrary, GSH level and GSH/GSSG ratio were further up-regulated and GSSG level was sharply down-regulated by pretreatment of ZEA in ZEA + Aβ groups (Figure 4).

### The Rectification of Zeaxanthin on Endothelin-1 Expression in Plasma

**FIGURE 5:**
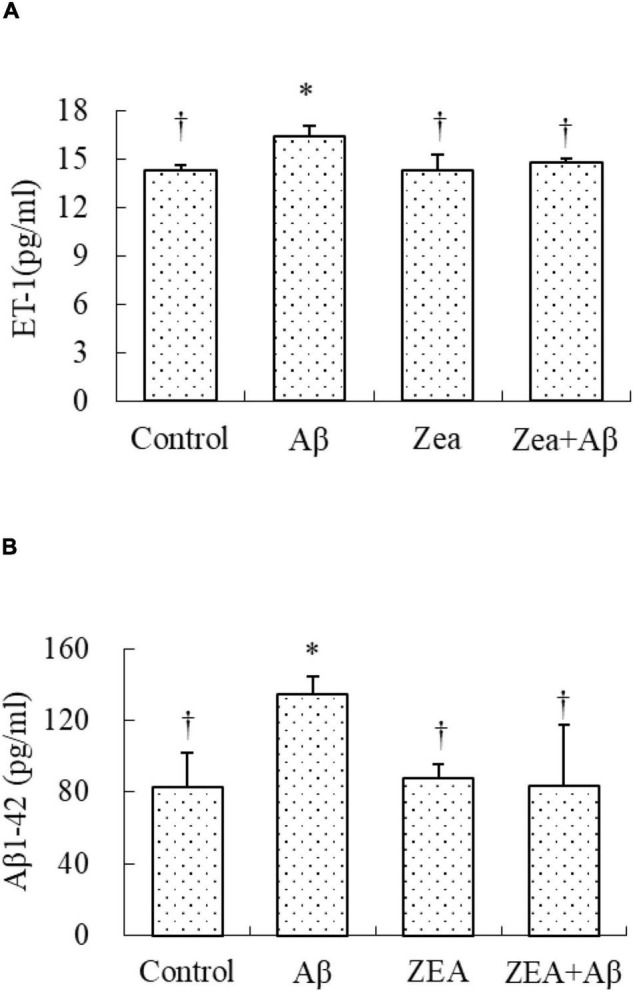
Neuroprotective effects of ZEA on the level of ET1 **(A)** and the level of Aβ1–42 **(B)** in plasma of learning and memory impairment rats induced by Aβ1–42. ELISA was used in the rat plasma from four treatment groups mentioned before (*n* = 12 per group). All the data were shown as mean ± SD, and analyzed by the one-way ANOVA test [*post hoc* (LSD) test]. *Significantly different from the Control group (*p* < 0.05). ^†^Significantly different from the Aβ group (*p* < 0.05).

Because the damage to cerebrovascular tissue had been found in Figure 3, the ET-1 level was tested in this research to check the vascular protective effects of ZEA. The results showed that the ET-1 expression in plasma was significantly increased in the Aβ group, but it was markedly decreased in the ZEA + Aβ group (Figure 5A).

### The Regulation of Zeaxanthin on the Level of Aβ1–42

**FIGURE 6:**
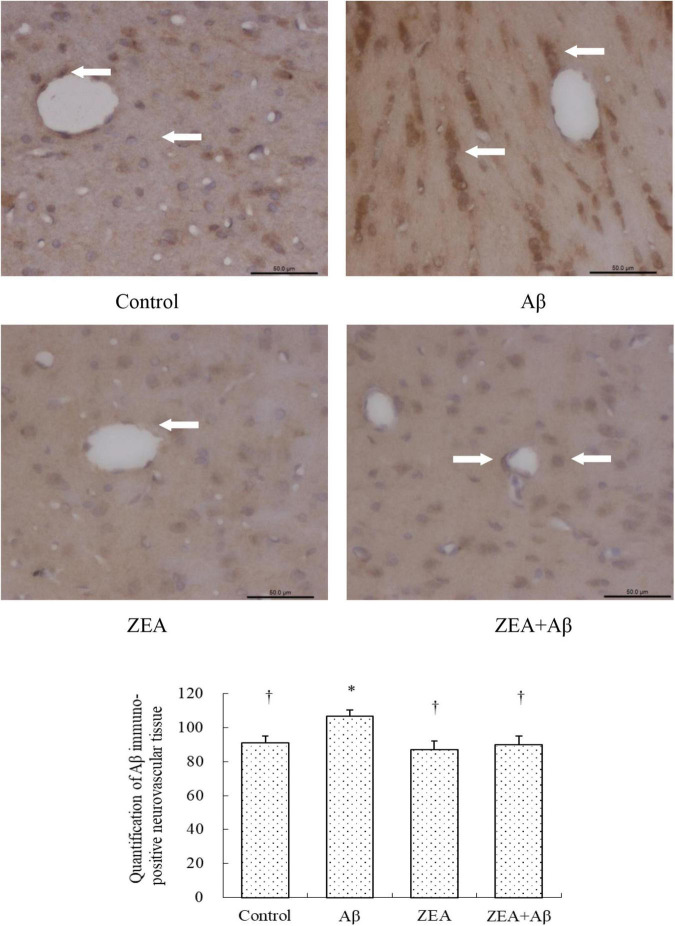
Neuroprotective effects of ZEA on Aβ in the cerebrovascular tissue of learning and memory impairment rats induced by Aβ1–42. Immunohistochemistry was used in the paraffin section of the whole rat brain from four treatment groups mentioned before (*n* = 12 per group). All the data were shown as mean ± SD, and analyzed by the one-way ANOVA test [*post hoc* (LSD) test]. *Significantly different from the Control group (*p* < 0.05). ^†^Significantly different from the Aβ group (*p* < 0.05). Staff gauge showed 100 μm at the right side of every pictures’ bottom.

Since Aβ in the central nervous system could be transported by the cerebrovascular system, we tried to investigate whether ZEA could decrease the level of Aβ1–42 in plasma, and explored the amyloidosis of tissue in different groups. As shown in Figure 5B, the Aβ1–42 level in plasma was obviously increased in the rats of the Aβ groups compared with that in the control group. It was also confirmed that the Aβ1–42 level in the rats of the ZEA + Aβ group was significantly decreased than that in the Aβ group. The changing trend in cerebrovascular was similar to that in plasma, Aβ1–42 expression in cerebrovascular tissue was significantly down-regulated in the ZEA + Aβ group than in the Aβ group (Figure 6).

### The Modification of Zeaxanthin on Aβ Transport Receptors and Inflammatory Cytokine in Cerebrovascular Tissue

**FIGURE 7:**
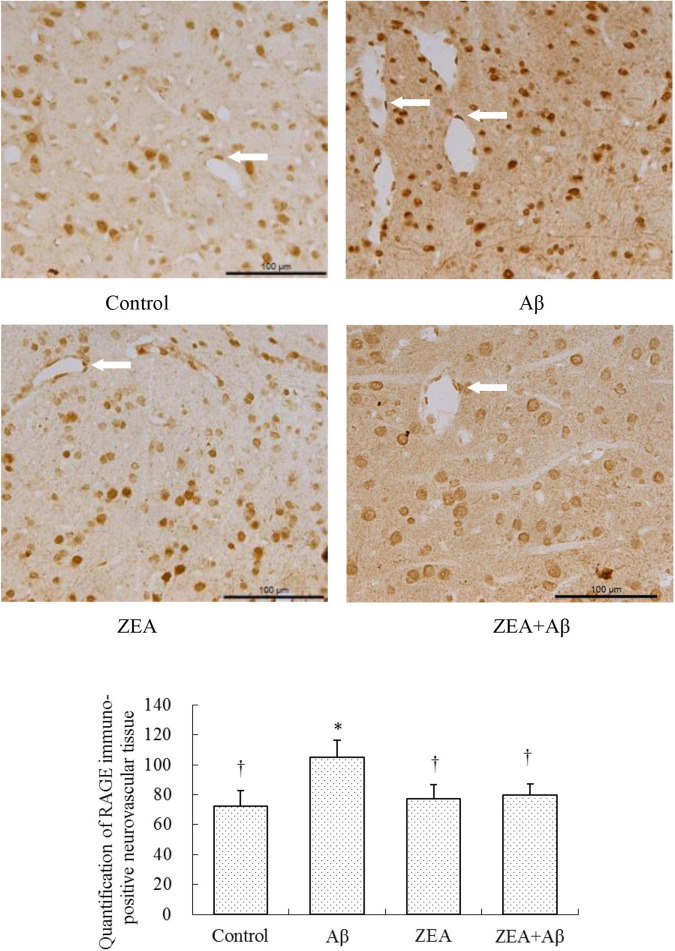
Neuroprotective effects of ZEA on RAGE in the cerebrovascular tissue of learning and memory impairment rats induced by Aβ1–42. Immunohistochemistry was used in the paraffin section of the whole rat brain from four treatment groups mentioned before (*n* = 12 per group). All the data were shown as mean ± SD, and analyzed by the one-way ANOVA test [*post hoc* (LSD) test]. *Significantly different from the Control group (*p* < 0.05). ^†^Significantly different from the Aβ group (*p* < 0.05). Staff gauge showed 100 μm at the right side of every pictures’ bottom.

**FIGURE 8:**
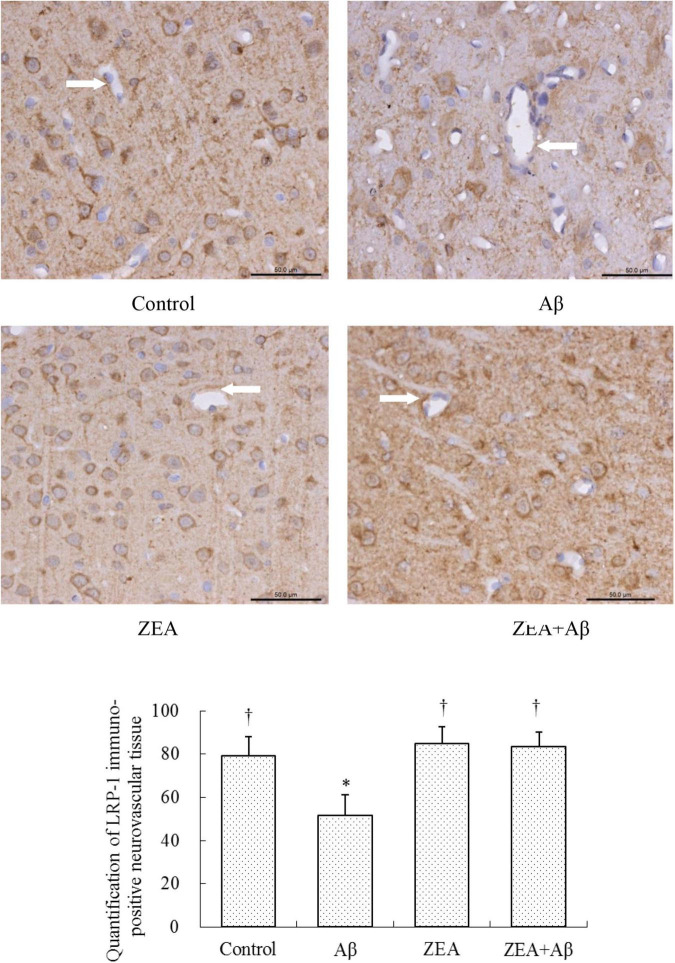
Neuroprotective effects of ZEA on LRP-1 in cerebrovascular tissue of learning and memory impairment rats induced by Aβ1–42. Immunohistochemistry was used in the paraffin section of the whole rat brain from four treatment groups mentioned before (*n* = 12 per group). All the data were shown as mean ± SD, and analyzed by the one-way ANOVA test [*post hoc* (LSD) test]. *Significantly different from the Control group (*p* < 0.05). ^†^Significantly different from the Aβ group (*p* < 0.05). Staff gauge showed 50 μm at the right side of every pictures’ bottom.

**FIGURE 9:**
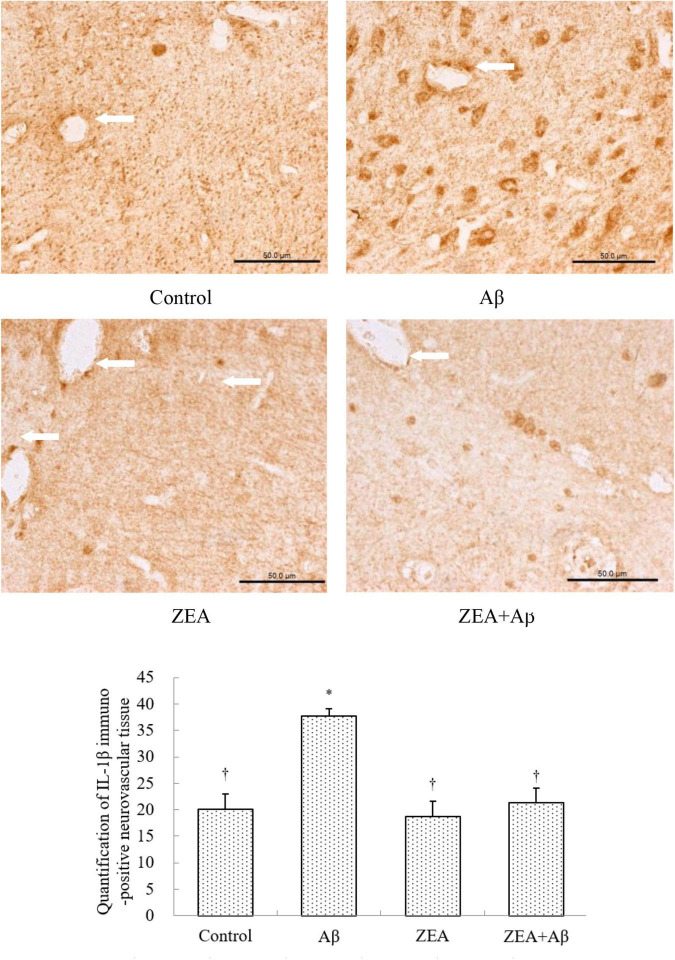
Neuroprotective effects of ZEA on IL-1β in cerebrovascular tissue of learning and memory impairment rats induced by Aβ1–42. Immunohistochemistry was used in the paraffin section of the whole rat brain from four treatment groups mentioned before (*n* = 12 per group). All the data were shown as mean ± SD, and analyzed by the one-way ANOVA test [*post hoc* (LSD) test]. *Significantly different from the Control group (*p* < 0.05). ^†^Significantly different from the Aβ group (*p* < 0.05). Staff gauge showed 50 μm at the right side of every pictures’ bottom.

To investigate the mechanism of ZEA against the cerebrovascular damage of Aβ, RAGE, and LRP-1, as the Aβ transport receptors in the cerebrovascular system were detected. The results showed that Aβ1–42 leads to the up-regulation of RAGE expression and down-regulation of LRP-1 expression in cerebrovascular tissue. However, ZEA pretreatment could reverse both these two receptors’ disordered expressions (Figures 7, 8). Compared with the control group, IL-1β protein expression in the neurovascular tissues was significantly up-regulated by the stimulation of Aβ1–42, but it was down-regulated in the ZEA + Aβ group (Figure 9).

## Discussion

Mild cognitive impairment (MCI) is the initial event of AD. Nearly, 10–15% of patients with MCI will convert into AD (Chechko et al., 2014). It is clear that Aβ could contribute to the reduction of learning and memory ability in patients with AD (Qiang et al., 2017). There is no controversy that vascular pathology and cerebrovascular dysfunction in the development of AD can be related to cognitive decline (Mataró et al., 2014). But, previous studies still have a debate, and more studies are needed to explore the relationship among Aβ, cerebrovascular dysfunction, and learning and memory impairment happened in MCI (Chong et al., 2019). In addition, numerous studies indicate that many functional foods such as ascorbic acid, vitamin E, and carotenoids have beneficial effects on cognitive health. ZEA is one kind of phytochemistry that has the capability of anti-oxidation. It is reported that ZEA could play an anti-oxidative role in ameliorating nervous diseases (Zhou et al., 2018). A variety of evidence support a key protective effect of lutein and ZEA in vision (Woodside et al., 2015; Wang et al., 2018; Ge et al., 2020; Monjotin et al., 2022). But, ZEA’s role in cognition has only been considered recently. Therefore, the beneficial effects of ZEA on learning and memory impairment need to be investigated. In addition, the relationship between cerebrovascular protection of ZEA and Aβ transportation needs to be further explored. In this study, ZEA pre-treatment showed the preventive effect on regulating the impairment of learning and memory ability of rats induced by Aβ1–42. This neuroprotection might be attributable to the anti-oxidative effects of ZEA on cerebrovascular defense. The mechanism might be associated with keeping the redox state balanced and reducing the Aβ level through regulating Aβ transport receptors in cerebrovascular tissue.

AD is a kind of neurodegenerative disease with symptoms of problems with language, mood swings, disorientation, and a series of behavioral issues because of learning and memory impairment (Arenaza-Urquijo et al., 2015). It has been reported that lycopene could rectify learning and memory deficits induced by Aβ1–42 in a dose-dependent manner (Sachdeva and Chopra, 2015). Our prior studies indicate that soybean isoflavone (SIF) could prevent cognitive impairment through its anti-oxidative and anti-inflammatory effects (Xi et al., 2013). The analogous results were also found in this study, the Morris water maze test indicated that ZEA could obviously shorten the escape latency of rats that was prolonged by Aβ1–42. And ZEA also could promote the frequency that rats swim across the position of the platform. These data implied that ZEA might play a protective role in cognitive impairment induced by Aβ. This neuroprotective effect is similar to the other functional food such as Epigallocatechin gallate in the tea polyphenol (Afzal et al., 2015).

In order to find out how ZEA conducts neuroprotection, cerebrovascular protective effects were explored. Ultrastructure of cerebrovascular tissue was first observed in different groups. The results showed that the cerebrovascular tissue was damaged by Aβ1–42 seriously, but ZEA prevented the morphological changes of cerebrovascular endothelial cell and the mitochondria injury in cerebrovascular tissue. This efficiency of ZEA was similar to SIF that had been reported by others and our previous studies (Xi et al., 2014, 2015). In the research of human retinal pigment epithelial cells, ZEA also showed cell protection against hypoxia-induced damage (Rosen et al., 2015). Therefore, the damage to cerebrovascular tissue combined with the results of the Morris water maze implied that the harm of Aβ1–42 on cognition might be associated with its damage to the neurovascular system cell. However, the learning and memory impairments and cerebrovascular injuries of rats could be revised by pre-treatment of ZEA.

To further investigate the reason for ZEA on the cerebrovascular protections against Aβ, the redox state of rats was investigated in this study. It is confirmed that Aβ-induced cerebrovascular endothelial cells’ oxidative damage could lead to endothelial injury and cerebrovascular dysfunction (Liu et al., 2015). GSH/GSSG, as the sensitive index of the redox state, is always used to determine the oxidative damage of rats. It is reported that GSH and GSSG levels in the hippocampus and cerebral cortex could be regulated by curcumin in a mouse model of AD (Sarker et al., 2015). There are studies demonstrating that genistein could reverse the disorder of GSH/GSSG in PC12 cells and cerebrovascular endothelial cells induced by Aβ (Xi et al., 2012; Qiang et al., 2017). Moreover, recent research shows that plasma total carotenoid levels (containing zeaxanthin, lutein, β-cryptoxanthin, α-carotene, β-carotene, and lycopene) in patients with chronic obstructive lung diseases (COPDs) are lower than these in the healthy elderly people. Homoplastically, GSH content in the blood of subjects of COPD was significantly lower than that in the healthy elderly (Kodama et al., 2017). Similar results were also found in this study, ZEA showed anti-oxidative effects through increasing GSH level and GSH/GSSG ratio and decreasing GSSG level in plasma which was disturbed by Aβ1–42. These results infer that the cerebrovascular protection of ZEA might be related to its anti-oxidative capability.

To check the vascular protection of ZEA, ET-1 level which was commonly used for clinical determination and analysis of vascular endothelial function was detected in plasma. There is evidence that ET-1 is involved in the pathogenesis of endothelial dysfunction induced by Aβ (Alcendor, 2020). In our results, Aβ could lead to a higher level of ET-1 and ZEA could dramatically cause a reduction of ET-1 in plasma. It is reported that ET-1 could decrease cerebral blood flow of its vasoconstrictor properties (Lei et al., 2015). Similar research manifests that treatment of lutein could show a strong neuroprotective effect against transient cerebral ischemic injury. They also demonstrate that this effect might be associated with the antioxidant property of lutein including the regulation of GSH/GSSG (Sun et al., 2014). Moreover, cerebral hypoperfusion could impair neuronal function, reduce the clearance of Aβ peptide and up-regulate Aβ production (Alcendor, 2020). In this case, we hypothesized that ZEA could protect the cerebrovascular system *via* its oxidative capacity. This effect might have some relationship with modulating Aβ level and Aβ clearance.

With the aim of finding the mechanism of ZEA preventing cerebrovascular dysfunction and cognitive defects, Aβ levels in plasma and cerebrovascular, and Aβ transport receptors in the cerebrovascular tissue were tested. Aβ homeostasis in humans is keeping a balance between production and proteolytic degradation. It is known that cerebrovascular cells of BBB are charged with the clearance of different forms of Aβ between the brain and blood circulation (Lin et al., 2022). Numerous evidence show that the microvasculature damages including the disordered expression of Aβ transport receptors such as RAGE and LRP-1 are found in the AD brains (Liu et al., 2012; Solis et al., 2020). RAGE and LRP-1 are considered a couple of crucial transporters of Aβ in cerebrovascular wall cells. RAGE mediates Aβ transporting into the brain from circulation and LRP-1 regulates Aβ pumping out of the brain (Lee et al., 2012). Our previous study demonstrates that SIF could reverse disordered Aβ homeostasis in rats. Simultaneously, SIF also could regulate RAGE and LRP-1 expressions through its anti-oxidative capability (Xi et al., 2013). It is also shown that lycopene could be used for the therapy of vascular inflammation by down-regulating the expression of RAGE and blocking the activation of pro-inflammatory cytokines (Lee et al., 2012). In this study, a higher level of Aβ1–42 in plasma and cerebrovascular found in rats of the Aβ group showed Aβ homeostasis was broken in the animal model, but pre-treatment of ZEA could keep Aβ1–42 at a steady and normal level in both plasma and cerebrovascular. Moreover, Aβ could lead to an up-regulated expression of RAGE and down-regulated expression of LRP-1 in cerebrovascular tissue, but ZEA could significantly rectify these two abnormal receptors. In order to investigate whether ZEA can protect neurovascular injury from RAGE-mediated neurovascular inflammation, the protein expression of inflammatory cytokine IL-1β was measured. The results manifested that ZEA could decrease the up-regulation of IL-1β protein expression induced by Aβ1–42. The findings suggest that ZEA might promote Aβ clearance through regulating expressions of RAGE, LRP-1, and IL-1β that are related to the oxidative damage and inflammation of cerebrovascular tissue.

## Conclusion

Aβ could impair the learning and memory ability of rats *via* oxidative damage and inflammation. Aβ overproduction and clearance dysfunction which could lead to the cerebrovascular dysfunction may play important role in cognitive damage. ZEA could prevent Aβ-induced cognitive impairment. This protective effect might be attributed to resisting oxidative damage and inflammation of the cerebrovascular tissue. The cerebrovascular protection of ZEA may be related to reducing the Aβ level in the brain and blood circulation by activating the Aβ clearance mechanism within circuit-specific pathways. The key targets of ZEA on Aβ transportation might be associated with the regulation of RAGE and LRP-1 expressions in the cerebrovascular tissue.

## Data Availability Statement

The original contributions presented in this study are included in the article/supplementary material, further inquiries can be directed to the corresponding author.

## Ethics Statement

The animal study was reviewed and approved by the Chinese Committee of Experimental Animal Supervision (Reg. No. AEEI-2014-047).

## Author Contributions

XL contributed to the study design, animal work, sample collection, data analysis, supervision of data collection, and writing the manuscript. PZ contributed to the data analysis, supervision of data collection, and writing the manuscript. HL and HY contributed to the data analysis and writing the manuscript. YX conceived the study question and contributed to the study design. All authors contributed to the article and approved the submitted version.

## Conflict of Interest

The authors declare that the research was conducted in the absence of any commercial or financial relationships that could be construed as a potential conflict of interest.

## Publisher’s Note

All claims expressed in this article are solely those of the authors and do not necessarily represent those of their affiliated organizations, or those of the publisher, the editors and the reviewers. Any product that may be evaluated in this article, or claim that may be made by its manufacturer, is not guaranteed or endorsed by the publisher.
